# Culturally adapted wearable mobile health intervention for physical activity and cognitive function in middle-aged Koreans: a pilot feasibility study

**DOI:** 10.12701/jyms.2026.43.53

**Published:** 2026-08-10

**Authors:** Woo-young Shin, Seungju Baek, Sei Young Lee, Changwon Lim, Kwangsu Moon, Sunmee Jang, Jung-ha Kim

**Affiliations:** 1 Department of Family Medicine, Chung-Ang University Medical Center, Chung-Ang University College of Medicine, Seoul, Korea; 2 Department of Nursing, Graduate School of Chung-Ang University, Seoul, Korea; 3 Department of Otorhinolaryngology-Head and Neck Surgery, Chung-Ang University College of Medicine, Seoul, Korea; 4 Department of Applied Statistics, Chung-Ang University, Seoul, Korea; 5 Department of Psychology, Chung-Ang University, Seoul, Korea; 6 College of Pharmacy, Gachon University, Incheon, Korea

**Keywords:** Cognition, Culturally competent care, Exercise, Middle aged, Telemedicine

## Abstract

**Background:**

Middle adulthood (45–64 years) is a critical window for the primary prevention of cognitive decline and chronic diseases, and physical activity can reduce later cognitive decline by 20% to 35%. However, most evidence on mobile health (mHealth) interventions for physical activity comes from Western populations, with few evaluations of culturally adapted approaches for Asian middle-aged adults, particularly in rapidly aging South Korea.

**Methods:**

This 12-week, single-arm, pre-post pilot feasibility study enrolled 304 community-dwelling adults aged 45–64 years from Dobong-gu, Seoul, South Korea. The intervention combined a Samsung Galaxy Watch 6 with a custom application incorporating culturally adapted features (mountain-climbing gamification based on famous Korean peaks, adaptive goal setting, and context-aware notifications) developed through systematic focus groups. The primary outcomes were weekly step count and moderate-to-vigorous physical activity (MVPA); secondary outcomes included subjective cognitive function (validated self-report scale), exercise behavior stages, depressive symptoms, and app usability.

**Results:**

Overall, 302 participants (99.3%) completed the intervention. Weekly step count increased by 16.9% (78,334 to 91,531; *p*<0.001) and weekly MVPA by 83.3% (56.0 to 102.6 minutes; *p*<0.001). The proportion meeting World Health Organization physical activity guidelines increased from 23.2% to 42.1% (*p*<0.001). Selective improvements occurred in executive function-related cognitive domains and depressive symptoms, and a dose-response relationship emerged between app engagement and step count gains.

**Conclusion:**

This culturally adapted wearable mHealth intervention showed high feasibility and acceptability for promoting physical activity in Korean middle-aged adults, supporting further evaluation as a scalable strategy for the primary prevention of cognitive decline and chronic diseases.

## Introduction

Population aging is accelerating globally and the proportion of adults aged 65 years and older is projected to increase from 8% to 16% by 2050 [1]. East Asian countries are aging rapidly, with South Korea achieving super-aged society status in 2024, faster than any other nation [2]. This rapid transition has prioritized the development of effective interventions for healthy aging.

Middle age (45–64 years) represents a critical window for intervention. During this period, biological aging accelerates and risk factors for chronic disease and cognitive decline emerge; however, individuals remain highly responsive to lifestyle modifications [3]. Physical activity during middle age can reduce the risk of subsequent cognitive decline by 20% to 35% through enhanced neuroplasticity and improved cerebrovascular function [4]. Investigating cognitive health in midlife is essential for identifying early windows to delay clinical impairment. Recent longitudinal evidence indicates that initiating moderate-to-vigorous physical activity (MVPA) during this period significantly reduces cognitive decline, likely through enhanced neuroplasticity and cerebrovascular function [5,6]. Consequently, targeting physical activity as the primary outcome, with cognition as a secondary outcome, provides a robust framework for evaluating the effectiveness of early-stage preventive mobile health (mHealth) interventions. However, physical inactivity remains highly prevalent among middle-aged adults worldwide, highlighting the need for effective and sustainable intervention strategies [7]. In rapidly aging societies, sedentary occupational demands, time constraints, and limited recreational opportunities further compound these risks, underscoring the need for accessible and culturally relevant intervention models that can shift physical activity trajectories before age-related decline accelerates.

mHealth interventions that leverage wearable technology have demonstrated effectiveness in promoting physical activity, with evidence suggesting activity increases of 12% to 25% [8]. These interventions typically incorporate evidence-based behavior change techniques such as goal setting, self-monitoring, and personalized feedback [9]. However, most research has been conducted on Western populations, with limited evidence from Asian populations, where cultural values, health beliefs, and technology adoption patterns may substantially influence intervention effectiveness [10]. Furthermore, few studies have explicitly designed interventions with cultural adaptation as the core principle, despite the growing recognition that this factor is crucial for engagement and adherence.

This study evaluated the feasibility, acceptability, and preliminary effectiveness of a culturally adapted mHealth intervention, designed to promote physical activity and cognitive function in middle-aged Korean adults. We hypothesized that systematic cultural adaptation would result in high retention of and meaningful improvements in physical activity and cognitive outcomes.

## Methods

**Ethics statement:** This study was approved by the Institutional Review Board (IRB) of Chung-Ang University (IRB No: 1041078-20240131-BR-020) and adhered to the Declaration of Helsinki. All participants provided written informed consent prior to enrollment. The participants were compensated up to 400,000 Korean won (approximately US$290) for their time, study assessments, and continuous wearing of the device over the 13-week study period.

### 1. Study design and participants

This was a 13-week, single-arm, pre-post pilot feasibility study comprising a baseline assessment week (week 0) and a 12-week intervention period (weeks 1–12), conducted from May 2024 to October 2024. Community-dwelling middle-aged Korean adults aged 45 to 64 years were recruited to evaluate a culturally adapted mHealth intervention. This age range is widely recognized in epidemiological research as middle adulthood or the critical transition period preceding later life [11]. The upper age limit was set at 64 years to focus on the transitional period prior to the legal definition of ‘older adults’ (≥65 years) under the South Korean Welfare of Senior Citizens Act [12]. The single-arm design was selected as an initial step to establish feasibility and preliminary effectiveness, following the United Kingdom Medical Research Council framework for complex interventions, which recommends pilot studies before proceeding to a definitive randomized controlled trial [13]. The 12-week duration was chosen based on behavioral evidence indicating that 12 to 16 weeks is the optimal period for habit formation [14].

Participants were recruited from Dobong-gu, a residential urban district in northern Seoul, South Korea, with a population of 303,228 as of 2024, 24.5% of whom were ≥65 years of age. With one of the highest aging rates among the 25 districts of Seoul (citywide average ≥65 years of age: 19.4%), Dobong-gu is classified as a super-aged society, which provides a critical context for interventions targeting middle-aged and older adults [15]. Participant recruitment was conducted in collaboration with Dobong-gu and the Dobong-gu Medical Association. Initial enrollment was facilitated by three Dobong-gu-based primary care clinics. Additional participants were subsequently recruited through snowball sampling via participant referrals to ensure that all referred individuals met the predefined eligibility criteria.

The inclusion criteria were as follows: (1) age 45 to 64 years, (2) resident or employee in Dobong-gu, (3) owner of an Android smartphone (operating system version 13.0 or higher), (4) ability to wear a smartwatch continuously, and (5) negative responses on the Physical Activity Readiness Questionnaire (PAR-Q) or physician clearance for physical activity if responses were positive. The exclusion criteria included functional limitations preventing independent ambulation, active wrist skin conditions, a history of severe phlebotomy reactions, cognitive impairment that would prevent informed consent, planned extended travel during the study period, and current participation in another intervention study.

### 3. Intervention

The intervention was delivered through a Samsung Galaxy Watch 6 integrated with a custom-built ‘Active Plan’ mobile application comprising five core components: (1) real-time activity monitoring with visual feedback, (2) adaptive daily step goals set approximately 500 steps above the previous week’s average, (3) context-aware motivational notifications, (4) automated MVPA detection using heart rate reserve calculations with 10-minute bout requirements, and (5) gamification featuring a culturally resonant mountain-climbing theme progressing through five levels (Supplementary Material 1) [16]. The intervention design was grounded in established behavior change frameworks, including the transtheoretical model and the capability, opportunity, motivation–behavior (COM-B) framework. It was further refined for cultural relevance through systematic focus groups and usability testing with middle-aged Korean adults (Supplementary Material 2) [17].

All participants attended a baseline visit for standardized device setup, including guided pairing of the smartwatch and app installation. Technical support was provided throughout the intervention. Participants were instructed to wear the smartwatch continuously (24 hours per day) throughout the study period, removing the device only for charging or when contraindicated (e.g., water exposure exceeding device specifications). To support valid measurements, participants were encouraged to aim for at least 10 hours of wear per day, consistent with the commonly applied wear-time guidance for accelerometer-based physical activity monitoring.

### 4. Outcomes and measures

Study outcomes were assessed across physical, cognitive, behavioral, and psychosocial domains using validated instruments. The individual instruments and their citations are described in the corresponding subsections below. The primary outcomes were physical activity measures. Physical activity was continuously monitored via the smartwatch, capturing key metrics aligned with World Health Organization (WHO) guidelines, including weekly step count, daily average steps on valid wear days (≥1,000 steps to exclude non-wear periods), and weekly MVPA minutes [18]. Weekly MVPA minutes were calculated as the sum of moderate-intensity activity minutes plus double the vigorous-intensity minutes, consistent with WHO recommendations. Exercise intensity was determined using the heart rate reserve (HRR) method.


$$\mathrm{HRR}=\frac{\mathrm{measured}\;\mathrm{heart}\;\mathrm{rate}-\mathrm{resting}\;\mathrm{heart}\;\mathrm{rate}}{(220-\mathrm{age})-\mathrm{resting}\;\mathrm{heart}\;\mathrm{rate}}\times 100$$

Intensities of 60% to 85% were defined as moderate and >85% as vigorous. Resting heart rate was defined as the lowest value recorded between 03:00 AM and 07:00 AM each day to establish an individual baseline [19]. A key categorical outcome was the proportion of participants meeting WHO physical activity guidelines (≥150 minutes of MVPA per week). Cognitive function, a key secondary outcome, was evaluated using the Subjective Cognitive Function Decline Scale for Middle-aged Koreans (SCFD-K), a 20-item, culturally specific instrument in which lower scores indicate better subjective function [20]. The Korean-language version of the SCFD-K used in this study is provided in Supplementary Material 3.

Other secondary outcomes included exercise behavior stage, assessed using a culturally adapted transtheoretical model questionnaire; psychosocial factors, including depressive symptoms measured by the 10-item Center for Epidemiologic Studies Depression Scale (CESD-10); and subjective health status evaluated using a single 5-point Likert scale [21,22]. The outcomes were assessed at baseline (week 0) and postintervention (week 12). Physical activity was monitored during the baseline assessment week and throughout the intervention period, and all pre-post analyses compared data collected at week 0 and week 12. App usability was assessed postintervention using a 42-item questionnaire to evaluate functionality, engagement, and design. The questionnaire was adapted from the Mobile Application Rating Scale and previously published instruments for mobile app usability; its full item list is provided in Supplementary Material 4.

### 5. Data collection

Baseline assessments were conducted during a 90-minute visit to a physician-led community care integration center. The participants completed electronic questionnaires via the Active Plan app to obtain sociodemographic, health status, and psychosocial data. During the 13-week period, physical activity data were automatically synchronized daily; for failures exceeding 48 hours, the research staff contacted participants via text or phone calls to facilitate syncing. Device-wear adherence was assessed as the proportion of valid wear days (≥1,000 steps) out of the total study days. Postintervention assessments, including the usability questionnaire, were completed within 1 week of the conclusion of the intervention. The 48-hour threshold referred to consecutive synchronization failures; intermittent losses (e.g., single days of missed synchronization interspersed with successful synchronization) did not trigger participant contact, and such participants were retained in the analysis if they met the overall valid-wear-day criterion. Among the 302 participants included in the analysis, physical activity data met the prespecified completeness criteria (i.e., a sufficient number of valid wear days over the study period), and no additional completeness criterion was applied.

### 6. Statistical analysis

Sample size was calculated using G*Power 3.1.9.7 for a paired *t*-test, assuming a small effect size (d=0.20) based on a meta-analysis of mHealth interventions targeting physical activity in adults aged <65 years (Hedges’ g=0.281) and accounting for a 40% dropout rate, resulting in a target of 300 participants [23,24]. Analyses were performed using IBM SPSS ver. 28.0 (IBM Corp., Armonk, NY, USA) and R version 4.1.0 (R Foundation, Vienna, Austria). Normality was assessed using the Shapiro-Wilk test. Non-normally distributed continuous variables were analyzed using the Wilcoxon signed-rank test, with effect sizes calculated as r=Z/√n [25]; normally distributed variables were analyzed using paired *t*-tests. Categorical outcomes, such as the proportion meeting WHO guidelines, were analyzed using the McNemar test, and transitions between exercise behavior stages were assessed using the McNemar-Bowker test.

The analyses were based on complete pre- and post-data. Missing data were handled using outcome-specific complete-case analysis. Specifically, physical activity analyses included 302 participants who provided baseline (week 0) and postintervention (week 12) data. In contrast, cognitive and psychosocial outcomes were analyzed using all 304 participants because no missing values were present for these variables. Additionally, exploratory analyses were conducted to examine whether changes in primary outcomes differed according to educational level categories. As changes in weekly step counts and weekly MVPA were not normally distributed, between-group differences were examined using the Kruskal-Wallis test. Given the pilot nature of the study, analyses of secondary outcomes were considered exploratory and adjustments for multiple comparisons were not applied. Statistical significance was set at a two-tailed *p*<0.05 [26].

## Results

A total of 415 individuals were screened for eligibility between May 2 and July 10, 2024. After telephone prescreening, 307 individuals were deemed preliminarily eligible and underwent a baseline assessment. Three participants were excluded (one for positive PAR-Q results without physician clearance, one for an incompatible smartphone operating system, and one for withdrawal of consent), resulting in the enrollment of 304 participants. Two participants were excluded due to missing physical activity data, leaving 302 participants for the final analysis. The participants were predominantly female (63.2% [192/304]) and highly educated (57.9% [176/304] had a university degree or higher). The detailed baseline characteristics are presented in Table 1.

After the 13-week duration, the participants showed significant improvements in all physical activity measures (Table 2). The median weekly step count increased by 16.9%, from 78,334 to 91,531 steps (*p*<0.001), and weekly MVPA nearly doubled, increasing by 83.3%, from 56.0 to 102.6 minutes (*p*<0.001). Consequently, the proportion of participants meeting the WHO physical activity guidelines increased from 23.2% (70/302) to 42.1% (127/302) (*p*<0.001). Among the participants with complete data, 72.2% (218/302) showed an increase in weekly steps. Educational level was reclassified into three categories (high school or lower, n=127; college/university, n=158; and graduate school or higher, n=18). Exploratory analyses using the Kruskal–Wallis test suggested that changes in weekly step counts differed across educational level categories (*p*=0.045), whereas changes in weekly MVPA did not (*p*=0.799).

Device-wear adherence improved over the study period; the mean adherence increased from 81% at baseline to >95% from 6 weeks onward, with the median consistently at 100% (Fig. 1).

At baseline, 282 participants (92.8% [282/304]) scored below the SCFD-K clinical cutoff of 16 points, with 283 (93.1% [283/304]) remaining below the cutoff postintervention. The total SCFD-K score showed no significant change (*p*=0.576). Domain-specific analyses revealed significant improvements in language ability (*p*=0.030) and emotional regulation (*p*=0.009), whereas memory and visuospatial abilities showed no significant changes (Table 3).

There was a significant positive shift in exercise behavior stages (McNemar-Bowker *χ*²=28.681, *p*=0.001), with 77 participants (25.3% [77/304]) advancing to a higher stage of readiness for exercise. The proportion of regular exercisers increased from 61.8% (188/304) to 70.1% (213/304) (*p*<0.001). The psychosocial outcomes also improved. Depressive symptoms, as measured by CESD-10, decreased significantly (*p*=0.041), and the participants’ subjective health perception improved (*p*=0.006).

The overall usability score of the Active Plan app was 3.64 (standard deviation, 0.85) on a 5-point scale (Supplementary Material 4). A total of 272 participants (89.5% [272/304]) reported experiencing at least one technical error during the intervention, primarily related to synchronization or data loading. A dose-response relationship was observed between app engagement and outcomes. Participants in the highest tertile of app usage demonstrated a 23.4% greater increase in step count than those in the lowest tertile (*p*=0.008).

## Discussion

The results of this pilot study suggest that a culturally adapted mHealth intervention can achieve high feasibility and acceptability in promoting physical activity among middle-aged Korean adults, with 99.3% participant retention. The intervention yielded significant improvements in the primary outcomes; participants increased their median weekly step count by 16.9% and median weekly MVPA by 83.3%. The high retention contrasts with the typical mHealth retention rates of 50% to 70% reported in digital health studies [27,28], indicating that systematic cultural adaptation may contribute to addressing engagement barriers in digital health interventions.

Our multidomain assessment revealed potential secondary benefits beyond physical activity. We observed modest changes in selective cognitive domains related to executive function, specifically language ability and emotion regulation. These findings should be interpreted with caution as preliminary signals of feasibility rather than as definitive evidence of clinical efficacy. A dose-response relationship emerged, with participants in the highest tertile of app engagement showing 23.4% greater step count increases, providing evidence for the mechanism of action of the intervention. Regarding app engagement, although the mean usability score (3.64) is acceptable for a pilot study, the high rate of reported errors (89.5%) suggests that the current system requires significant technical refinement. These findings indicate that while the intervention is feasible, ensuring a more stable user experience is critical before proceeding to a definitive randomized controlled trial. Taken together, these findings suggest that culturally tailored mHealth platforms offer a scalable approach for promoting healthy aging in rapidly aging Asian societies.

The magnitude of physical activity improvements observed in this study is noteworthy when compared to reported values in existing literature. The average increase of approximately 1,885 steps per day exceeded the 700 to 1,300 steps typically reported in meta-analyses of wearable device interventions [29,30]. This effect size was statistically significant and approached the thresholds associated with reduced mortality risk in epidemiological studies [31]. This is notable given that our participants had a relatively high level of baseline activity, a context in which achieving further significant gains is often challenging [32]. Furthermore, the 83.3% increase in weekly MVPA represents a substantial improvement over the more modest changes often observed in digital health studies [33], indicating that the intervention not only increased overall movement but also promoted structured, purposeful exercise.

A retention rate of 99.3% was a key finding of this feasibility study. This figure substantially exceeds the 50% to 70% retention rates typically reported in mHealth research [27,28]. We hypothesize that this high engagement may be partly attributable to our systematic cultural adaptation process (Supplementary Material 2), which is consistent with the principle that user-centered and culturally resonant designs are crucial for effectiveness [34,35]. The dose-response relationship, in which higher app engagement correlated with greater increases in physical activity, provided additional evidence for the mechanism of action of the intervention. These findings suggest that cultural adaptation is an important factor for achieving sustained engagement in digital health interventions.

Our findings of selective improvements in cognitive function, specifically language ability and emotional regulation, align well with the established neuroscientific literature. Physical activity is known to have the most pronounced short-term effects on the prefrontal cortex, the brain region responsible for executive functions, including language and emotional control [36,37]. The fact that our intervention benefited from these executive-related domains while memory and visuospatial abilities remained unchanged is consistent with this neurobiological model. The lack of change in these domains suggests that more prolonged or higher-intensity exercise is required to induce detectable improvements, lending theoretical plausibility to our results. However, because cognitive function was assessed solely through a subjective self-report scale rather than objective neuropsychological testing, any inference linking these improvements to underlying neural mechanisms remains speculative and warrants confirmation in future trials using objective measures.

This study had several strengths. First, the large sample size (n=304) of this pilot study provided sufficient statistical power to detect meaningful changes and generate robust estimates for planning a definitive randomized controlled trial [26]. Second, this study was conducted in a real-world community-based setting in partnership with primary care clinics, thereby enhancing the external validity of our findings [38]. Third, the systematic cultural adaptation process is a core methodological strength that provides a replicable framework for developing interventions for diverse populations. Finally, our use of a multidomain outcome assessment allowed for a more holistic evaluation of the intervention’s overall impact. Implemented in collaboration with community-based primary care clinics and local government, this study also offers a transferable, community-embedded model for delivering scalable preventive digital health interventions to middle-aged populations at risk of physical inactivity and cognitive decline.

Despite these strengths, this study had some limitations that must also be acknowledged. The most significant is the single-arm, pre-post study design, which cannot establish causality and is susceptible to confounding factors such as seasonal effects, participant motivation, or the Hawthorne effect (i.e., the effect of repeated monitoring or device use alone) [39,40]. Second, the 12-week intervention period, which was sufficient for initial behavioral changes, did not provide evidence of long-term sustainability [41]. Third, our sample, composed of volunteers with a high level of education from an urban district, together with an exceptionally high retention rate (99.3%), may reflect a particularly motivated volunteer sample, introducing the possibility of selection bias and limiting generalizability to rural or less digitally literate populations [42]. Fourth, given the pilot and exploratory nature of this study, adjustments for multiple comparisons were not applied to secondary outcomes, which increased the risk of type I errors; therefore, the findings for secondary and domain-specific outcomes should be interpreted as preliminary. Finally, our cognitive function assessment relied on a subjective self-report scale. Although culturally validated, the absence of objective neuropsychological tests indicates that the observed improvements, although statistically significant, should be interpreted with caution. This highlights the need for future trials incorporating objective neuropsychological tests to validate these promising subjective findings.

This study provides preliminary evidence that a culturally adapted mHealth intervention is highly feasible and acceptable for promoting physical activity and shows the potential for supporting cognitive function in middle-aged Korean adults. The high retention rate and dose-response relationship underscore the value of our systematic, user-centered cultural adaptation approach, indicating that cultural adaptation may help address engagement challenges in digital health interventions. These promising findings warrant a definitive randomized controlled trial to establish causality and evaluate long-term sustainability. If confirmed, this scalable model could inform the development of effective strategies for promoting healthy aging in rapidly aging societies. Future research should assess its effectiveness in more diverse populations and incorporate objective cognitive measures to build on the findings of this pilot study.

## Figures and Tables

**Fig. 1.**
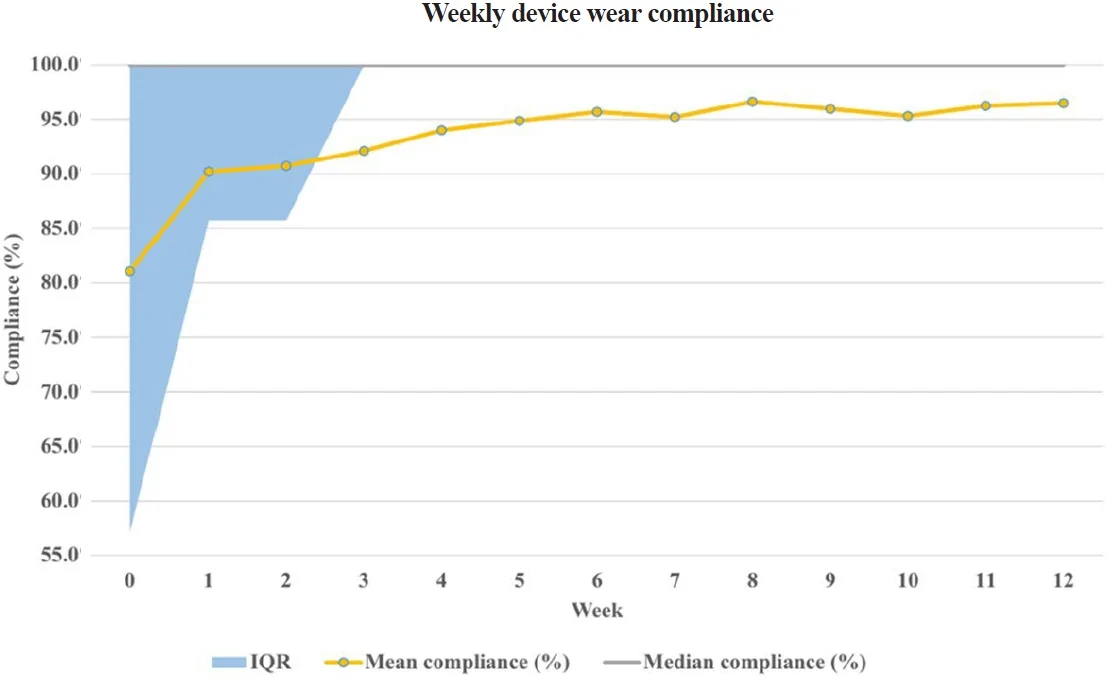
Weekly device wear adherence during the 12-week intervention period. Device wear adherence is defined as the proportion of valid wear days. Weekly adherence is calculated as the number of valid wear days (≥1,000 steps) divided by the total number of days in that week (i.e., a per-week wear-day percentage), rather than as the percentage of hours worn out of a 24-hour day. The solid yellow line indicates the mean adherence, the shaded area represents the interquartile range (IQR), and the gray line denotes the median. Adherence increases after the baseline week and stabilizes above 95% from week 6 onward, with the median remaining at 100% across all weeks.

**Table 1.** Baseline characteristics of the study participants (n=304)

Characteristic	Value
Age (yr)	
45–49	92 (30.3)
50–54	88 (29.0)
55–59	73 (24.0)
60–64	51 (16.8)
Sex	
Female	192 (63.2)
Male	112 (36.8)
Marital status	
Married or cohabiting	263 (86.5)
Never married	12 (4.0)
Separated, divorced, or widowed	29 (9.5)
Educational level	
High school or less	127 (42.1)
College or university	158 (52.0)
Graduate school or above	18 (5.9)
Chronic conditions	
Hypertension	117 (38.5)
Diabetes mellitus	62 (20.4)
Dyslipidemia	47 (15.5)
Cancer	25 (8.2)

Values are presented as number (%). Higher education was defined as completion of college or a higher level of education. Educational level was missing for one participant; therefore, the three categories sum to 303.

**Table 2.** Physical activity outcomes at baseline and after the 12-week intervention (n=302)

Outcome	Baseline	Postintervention	Change (%)	*p*-value^a)^	Effect size (r)
Weekly steps	78,334 (51,063–101,631)	91,531 (67,754–119,562)	+16.9	<0.001	0.335
Daily steps	12,800 (9,871–16,156)	13,182 (9,826–17,412)	+3.0	0.049	0.113
Weekly MVPA (min)	56.0 (20.60–143.29)	102.6 (44.5–266.8)	+83.3	<0.001	0.415
Meeting WHO guidelines^b)^	70 (23.2)	127 (42.1)	+18.9^c)^	<0.001^d)^	NA

Values are presented as median (interquartile range) or number (%) unless otherwise specified. MVPA, moderate-to-vigorous physical activity; WHO, World Health Organization; NA, not available.

a) All *p*-values were calculated using the Wilcoxon signed-rank test, except where noted.

b) WHO guidelines are defined as achieving ≥150 minutes of MVPA per week.

c) Percentage point difference.

d) Calculated using the McNemar test.

**Table 3.** Subjective cognitive function scores at baseline and after the 12-week intervention (n=304)

Outcome	Baseline	Postintervention	Change (%)	*p*-value^a)^	Effect size (r)
SCFD-K^b)^					
Total score	5.0 (3.00–9.00)	5.0 (2.00–9.00)	0	0.576	-
Language	1.0 (0.00–2.00)	1.0 (0.00–2.00)	0	0.030	0.125
Visuospatial	0.0 (0.00–1.00)	0.0 (0.00–1.00)	0	0.548	-
Emotional	2.0 (0.00–3.00)	1.0 (0.00–3.00)	–50.0	0.009	0.150
Memory	2.0 (0.00–3.00)	2.0 (0.00–3.00)	0	0.612	-

Values are presented as median (interquartile range) unless otherwise indicated. SCFD-K, Subjective Cognitive Function Decline Scale for Middle-aged Koreans.

a) All *p*-values were calculated using the Wilcoxon signed-rank tests.

b) For the SCFD-K, lower scores indicate better subjective cognitive function. Total scores range from 0 to 60; each of four subdomains ranges 0–15. Clinical cutoff for suspected decline: total score ≥16. Observed ranges (minimum–maximum) at baseline and postintervention were as follows: Total, 0–30 and 0–28; Language, 0–10 and 0–8; Visuospatial, 0–10 and 0–7; Emotional, 0–8 and 0–9; Memory, 0–8 and 0–13.

## Supplementary materials

Supplementary Material 1. Key features of the Active Plan mobile application

Supplementary Material 2. Summary of focus group findings for cultural adaptation

Supplementary Material 3. Korean and English versions of the Subjective Cognitive Function Decline Scale for Middle-aged Koreans (SCFD-K) used in this study

Supplementary Material 4. Postintervention usability assessment of the Active Plan application (n=304)
