# Supplementary material for: Culturally adapted wearable mobile health intervention for physical activity and cognitive function in middle-aged Koreans: a pilot feasibility study
Source: J Yeungnam Med Sci. 2026 Aug 10;43:53. doi: 10.12701/jyms.2026.43.53 (PMC13681058; doi:10.12701/jyms.2026.43.53)
Supplement: Supplementary file 1 [file jyms-2026-43-53-Supplementary-Material-1.pdf]

Supplementary Material 1. Key features of the Active Plan mobile application

Feature	Description	Illustration (Korean UI screenshot with English captions)	
(1) Real-time activity monitoring	The participant's activity status was continuously tracked via the Galaxy Watch 6. Step counts and MVPA minutes were viewable through the smartphone app, which provided real-time feedback and visualizations of progress.	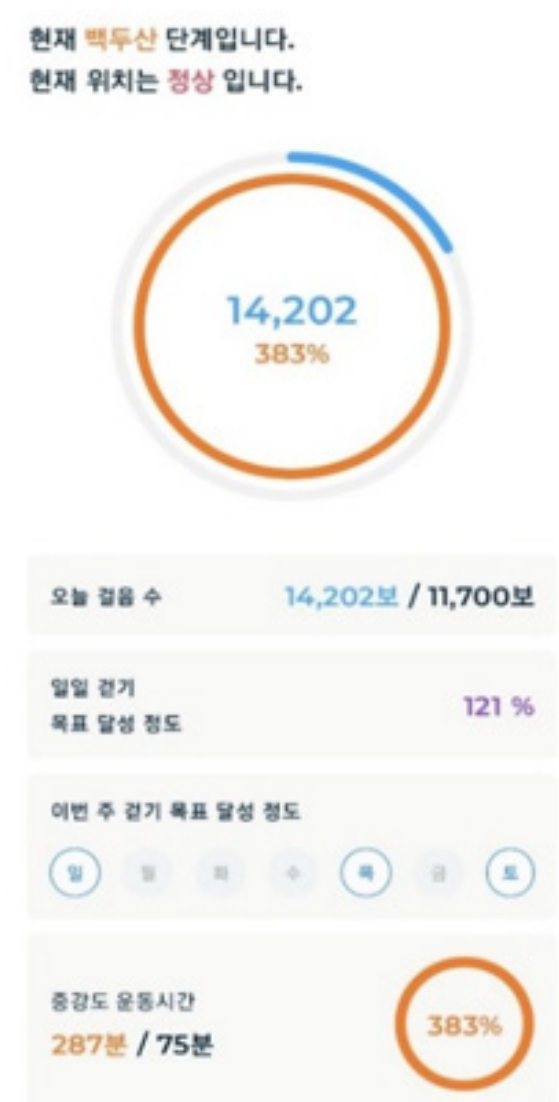	Displays steps, daily/weekly goal achievement, and MVPA minutes.
(2) Adaptive goal-setting	Participants were encouraged to manually set their daily step goals, typically 500 steps above their prior week's average. This manual input method was designed to enhance user autonomy.	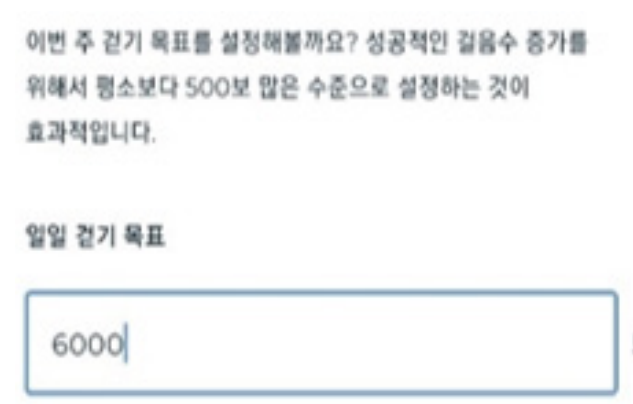	Daily walking goal input (e.g., 6000 steps).
(3) Context-aware motivational notifications	Personalized prompts and feedback messages were delivered to both the user's smartphone and Galaxy Watch 6 based on their goal achievement patterns to reinforce engagement and support behavior change.	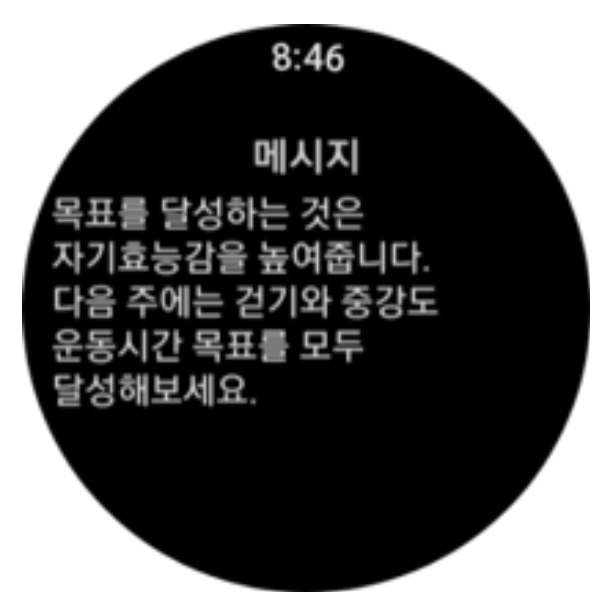	Motivational message encouraging goal completion.
(4) Automated MVPA detection	MVPA was automatically detected using the HRR method. To align with WHO guidelines, only bouts lasting 10 minutes or more were counted.	Same as Feature 1	
(5) Gamification (mountain-climbing theme)	To enhance engagement, participants progressed through five levels named after famous Korean mountains. Reviewing the previous week's activities and setting goals for the upcoming week allowed advancement to higher levels, where greater points could be earned, providing a culturally resonant sense of accomplishment.	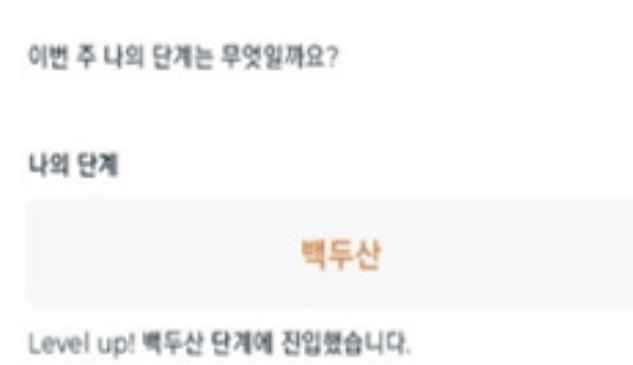	"Level up" to Baekdu Mountain stage.

UI, user interface; MVPA, moderate-to-vigorous physical activity; HRR, heart rate reserve.
Screenshots show the original Korean interface. English translations of key phrases are provided in captions for clarity.
