# Supplementary material for: Culturally adapted wearable mobile health intervention for physical activity and cognitive function in middle-aged Koreans: a pilot feasibility study
Source: J Yeungnam Med Sci. 2026 Aug 10;43:53. doi: 10.12701/jyms.2026.43.53 (PMC13681058; doi:10.12701/jyms.2026.43.53)
Supplement: Supplementary file 2 [file jyms-2026-43-53-Supplementary-Material-2.pdf]

Supplementary Material 2. Summary of focus group findings for cultural adaptation

Theme	Illustrative Participant Quote (Anonymous)	Resulting App Refinement
Gamification and Engagement		
(Participants favored non-competitive, personal achievement models over public rankings.)	"Competing with others is stressful, but I like the feeling of accomplishment, like I'm conquering Jirisan mountain by myself."	A personalized, non-competitive progression model based on climbing famous Korean mountains (e.g., Hallasan, Jirisan, Seoraksan) was adopted instead of a public leaderboard to foster a personal sense of achievement.
Goal Setting and Autonomy		
(Participants emphasized the importance of control and flexibility in setting their own goals.)	"Automatic goals are rigid. If I walked a lot yesterday, I want to rest a bit today. I want to set my own goals to feel in control."	The feature was changed from automatic adjustment to manual entry, allowing users to set their own daily goal (recommended: ~500 steps above the previous week's average) to enhance autonomy and self-regulation.
Motivational Message Framing		
(The perceived effectiveness of feedback depended on its framing, which varied by context and personality.)	"Just telling me I failed is demotivating. When I succeed, I want facts and data. When I miss a goal, I need some empathy or a rational reason to try again."	Message framing was tailored based on goal achievement and personality type (MBTI). Factual reinforcement and data-based feedback were used for success, while empathetic encouragement or loss-framed prompts were used for failure.
Information and Feedback		
(Participants valued accurate, real-time feedback but were easily overwhelmed by excessive information.)	"I value the accurate tracking, but sometimes there's too much information. I just want to know if I'm doing okay."	The user interface was streamlined to emphasize core tracking metrics (steps, MVPA) and minimize redundant data displays, focusing on clear, real-time feedback.
Message Content and Credibility		
(Participants distrusted generic messages and valued personalized, context-aware coaching.)	"Generic, robotic messages feel untrustworthy. I'm more likely to listen to personalized coaching that understands my situation."	Motivational prompts were made more context-aware and personalized to enhance credibility and engagement. Message frequency was also made flexible based on user preferences.

MBTI, Myers-Briggs type indicator.

Two focus group interviews were conducted with 14 middle-aged Korean adults (eight women, six men; mean age 54.2 years) to inform the cultural adaptation of the Active Plan app. The key themes, illustrative anonymous participant quotes, and resulting app refinements are summarized below.
