# Supplementary material for: Culturally adapted wearable mobile health intervention for physical activity and cognitive function in middle-aged Koreans: a pilot feasibility study
Source: J Yeungnam Med Sci. 2026 Aug 10;43:53. doi: 10.12701/jyms.2026.43.53 (PMC13681058; doi:10.12701/jyms.2026.43.53)
Supplement: Supplementary file 3 [file jyms-2026-43-53-Supplementary-Material-3.pdf]

Supplementary Material 3. Korean and English versions of the Subjective Cognitive Function Decline Scale for Middle-aged Koreans (SCFD-K) used in this study

No.	Korean item (원문)	English item
Language / 언어능력		
1	다른 사람에게 전달해야 할 메시지를 정확하게 표현하기 어렵다.	It is hard to articulate exactly what I need to convey to others.
2	상대방의 이야기나 글에서 핵심 내용을 파악하기 어렵다.	It is hard to get the gist of what the other person is saying or writing.
3	대화 중 내용이 이해되지 않아 반복해서 물어보곤 한다.	I do not understand something in a conversation, so I ask again.
4	책이나 기사를 읽을 때 같은 문장을 여러 번 반복해서 읽어야 이해가 된다.	When I read a book or article, I may have to read the same sentence repeatedly to understand it.
5	하고 싶은 말이나 표현이 금방 떠오르지 않는다.	I cannot think of the right words or expressions to say quickly.
Visuospatial / 시공간능력		
1	자주 가던 곳인데도 때때로 길을 헤맨다.	Sometimes I lose my way, even in places I have been before.
2	방향/공간 능력이 떨어져서 어느 쪽으로 가야 하는지 몰라 행동이 느려진다.	Orientation/spatial skills are decreased, and actions are slowed by not knowing which way to go.
3	설명을 들어도 가까운 장소를 찾아가는 것이 쉽지 않다.	Even with an explanation provided, it is not easy to find a place near me.
4	집 근처 도로나 골목길이 헷갈려서 잘못 들어갈 때가 있다.	Sometimes I get confused and turn into the wrong street or alley near my house.
5	약도나 내비게이션이 있어도 어디로 가야 할지 방향이 헷갈릴 때가 있다.	Even if I use a map or navigation, sometimes it is hard to determine where to go.
Personality/emotion / 성격·감정변화		
1	의욕적으로 하던 일들이 귀찮아지고 흥미가 줄어들었다.	The things I was motivated to do became a chore and I lost interest.
2	이전에는 활동적이었으나 점점 외출하기 싫고 집에만 있으려 한다.	I used to be active, but I increasingly do not want to go out and want to stay home.
3	전보다 사소한 일에 예민하게 반응하고 감정조절이 잘 안된다.	I am more sensitive to small things than before, and I cannot control my emotions.
4	이전에는 온화한 성격이었으나 최근 사나워지거나 욕설을 하는 등의 변화가 생겼다.	I used to be gentle, but recently I have become aggressive and abusive.
5	오랫동안 해 오던 일(직업, 취미, 집안 일)을 처리하는데 능력이 떨어진다고 느낀다.	I feel less capable of doing things I have been doing for a long time (work, hobbies, chores).
Memory / 기억력		
1	최근 들었던 이야기를 잊어버리고 같은 질문을 반복하곤 한다.	I forget what I have heard recently and repeat the same questions.
2	했던 말을 반복한다는 이야기를 듣곤 한다.	I have been told that I repeat what I have said.
3	요즘 들어 새로운 것(이름, 전화번호, 휴대폰/스마트폰 어플 사용법, 기기 작동법 등)을 기억하기 힘들다.	These days, it is hard to remember new information (names, phone numbers, how to use cell phone/smartphone apps, how to operate devices, etc.).
4	최근 소지품(휴대폰, 열쇠, 우산, 지갑 등)을 어디 두었는지 기억 못하거나 잃어버리곤 한다.	I cannot remember where I put my belongings (phone, keys, umbrella, wallet, etc.) lately, or I lose them.
5	어떤 일을 했다는 것을 잊어버려서 다시 그 일을 한 적이 있다.	I have forgotten that I did something, so I did it again.

SCFD-K, Subjective Cognitive Function Decline Scale for Middle-aged Koreans.
Each item is rated on a 4-point frequency scale scored 0–3: 0, not at all (<1 time/month; 전혀 아니다, 월 1회 미만); 1, sometimes (1–2 times/month; 가끔, 월 1~2회); 2, often (1–2 times/week; 자주, 주 1~2회); and 3, very often (3–4 times/week; 매우 자주, 주 3~4회). The scale comprises 20 items across four domains (5 items each). Each domain score ranges from 0 to 15 and the total score ranges from 0 to 60; lower scores indicate better subjective cognitive function.
The Korean items reproduce the validated final instrument; the English items are reproduced from So Y, Hahm J, Lee SY, Kim JH, Moon K. Development of the subjective cognitive function decline scale for middle-aged Koreans. Aging Ment Health 2025;29:906–14 (reference 20), under the Creative Commons License (CC BY-NC-ND 4.0).
