# Supplementary material for: Culturally adapted wearable mobile health intervention for physical activity and cognitive function in middle-aged Koreans: a pilot feasibility study
Source: J Yeungnam Med Sci. 2026 Aug 10;43:53. doi: 10.12701/jyms.2026.43.53 (PMC13681058; doi:10.12701/jyms.2026.43.53)
Supplement: Supplementary file 4 [file jyms-2026-43-53-Supplementary-Material-4.pdf]

Supplementary Material 4. Postintervention usability assessment of the Active Plan application (n=304)

Domain and Item Description	Mean	SD
1. Functionality		
1-1. Performance: Did the app's functions and components operate accurately and quickly?	3.50	1.13
1-2. Ease of use: Was it easy to learn how to use the app? Were the menus and icons intuitive?	3.86	0.96
1-3. Navigation: Was navigating between screens easy to understand and logical?	3.83	0.83
2. Engagement		
2-1. Entertainment: Were there fun or entertaining elements in using the app?	2.98	1.04
2-2. Interest: Did the app present information in an interesting way?	3.25	0.97
2-3. Appropriateness for target users: Was the app's content (e.g., visuals, language, design) appropriate for you?	3.83	0.79
3. Information & Design		
3-1. Information quality: Was the app's content well-written, accurate, and relevant?	3.66	0.83
3-2. Information quantity: Was the amount of information provided concise and appropriate?	3.92	0.96
3-3. Visual information: Were charts, graphs, and images displayed clearly and logically?	4.09	0.70
3-4. Source reliability: Did the app's information seem to come from reliable sources?	4.05	0.69
3-5. Layout: Was the most relevant content displayed first when accessing the app?	3.83	0.89
3-6. Visual placement: Were text and images placed in suitable locations?	4.00	0.72
3-7. Icon design: Was the app icon design appealing and familiar?	3.93	0.82
3-8. Readability: Was the text and other information arranged for high readability?	3.97	0.73
3-9. Conciseness: Was the information on the screen presented concisely with only essential information?	3.94	0.73
3-10. Color scheme: Was the number of main colors used appropriate and aesthetically pleasing?	3.64	0.84
4. Usability & User Control		
4-1. Information accuracy: Did the app accurately provide the information users wanted?	3.78	0.85
4-2. Simplicity: Was the process to view desired information convenient and not complex?	3.65	0.96
4-3. Menu structure: Were submenus arranged to logically match their parent menus?	3.73	0.77
4-4. Screen titles: Was a clear title presented on each screen?	3.94	0.68
4-5. Icon/Button quantity: Was the number of icons/buttons on each screen appropriate?	3.87	0.79
4-6. Menu clarity: Were all menus logical, clearly distinguished, and non-overlapping?	3.91	0.72
4-7. Guidance: Were directions or explanations appropriate for effective use of menus?	3.87	0.76
4-8. Convenience: Could you conveniently view desired information?	3.90	0.79
4-9. History access: Was it easy to find information you had previously viewed?	3.81	0.80
4-10. Ease of navigation: Was moving forward/backward and accessing other information simple and easy?	3.85	0.84
5. Error Management & Help		
5-1. Visual error notification: When errors occurred, was visual information provided?	2.95	1.27
5-2. Auditory error notification: When errors occurred, was auditory information provided?	2.63	1.21
5-3. Error message clarity: Were error messages concise and clear?	2.99	1.25
5-4. Instruction clarity: Were user instructions provided in an easy-to-understand visual manner?	3.81	0.74
5-5. Instruction accessibility: Was it convenient to access user instructions from any page?	3.72	0.80
5-6. Findability of instructions: Was there guidance to easily find instructions when needed?	3.66	0.87
6. Perceived App Quality & Effectiveness		
6-1. Step count accuracy: Do you think the step count was measured accurately?	3.64	0.97
6-2. Heart rate accuracy: Do you think the heart rate was measured accurately?	3.72	0.90
6-3. Data presentation: Were measured values and information well organized and provided?	3.95	0.76
6-4. Feedback effectiveness: Did the provided feedback help encourage more physical activity?	4.18	0.76
6-5. Goal-setting effectiveness: Did goal setting help encourage more physical activity?	4.08	0.90
6-6. Monitoring effectiveness: Did activity trend monitoring help encourage more physical activity?	4.16	0.79
7. Subjective Recommendation		
7-1. Recommendation: Would you recommend this app to others?	3.26	1.05
7-2. Willingness to pay: Would you be willing to pay to use this app?	2.76	0.90
7-3. Overall score: If you were to give this app an overall score?	3.58	0.72
Total Mean Score (All Items)	3.64	0.85

Postintervention usability was assessed using a 42-item questionnaire. The 41 items rated on a 5-point Likert scale (higher scores indicating a more positive assessment) are presented below, grouped by domain, with the mean and standard deviation (SD) for each item.

One survey item regarding error experience ("Did you experience any errors during pilot use?") was considered dichotomous (yes or no). As the results are described in the main text, they are excluded from this table which summarizes only the items rated on a 5-point Likert scale.
